# A novel compound heterozygous variant identified in *GLDC* gene in a Chinese family with non-ketotic hyperglycinemia

**DOI:** 10.1186/s12881-017-0517-1

**Published:** 2018-01-05

**Authors:** Yiming Lin, Zhenzhu Zheng, Wenjia Sun, Qingliu Fu

**Affiliations:** 1 Neonatal Disease Screening Center of Quanzhou, Quanzhou Women’s and Children’s Hospital, 700 Fengze Street, Quanzhou, Fujian Province 362000 China; 2 Genuine Diagnostics Company Limited, 859 Shixiang West Road, Hangzhou, Zhejiang Province 310007 China

**Keywords:** Non-ketotic hyperglycinemia, In silico, *GLDC* gene, Multiplex ligation-dependent probe amplification

## Abstract

**Background:**

Non-ketotic hyperglycinemia (NKH) is a rare, devastating autosomal recessive disorder of glycine metabolism with a very poor prognosis. Currently, few studies have reported genetic profiling of Chinese NKH patients. This study aimed to identify the genetic mutations in a Chinese family with NKH.

**Methods:**

A Chinese family of Han ethnicity, with three siblings with NKH was studied. Sanger sequencing and multiplex ligation-dependent probe amplification combined with SYBR green real-time quantitative PCR was used to identify potential mutations in the *GLDC*, *AMT* and *GCSH* genes. The potential pathogenicity of the identified missense mutation was analyzed using SIFT, PolyPhen-2, PROVEAN and MutationTaster software.

**Results:**

All patients exhibited severe and progressive clinical symptoms, including lethargy, hypotonia and seizures, and had greatly elevated glycine levels in their plasma and CSF. Molecular genetic analysis identified compound heterozygous variants in the *GLDC* gene in these three siblings, including a novel missense variant c.2680A > G (p.Thr894Ala) in exon 23 and a heterozygous deletion of exon 3, which were inherited respectively from their parents. In silico analysis, using several different types of bioinformatic software, predicted that the novel variant c.2680A > G in the *GLDC* gene was pathogenic. Moreover, the deletion of exon 3 was identified for the first time in a Chinese population.

**Conclusions:**

A novel missense variant and a previously reported deletion in *GLDC* gene were identified. The two variants of *GLDC* gene identified probably underlie the pathogenesis of non-ketotic hyperglycinemia in this family, and also enrich the mutational spectrum of *GLDC* gene.

**Electronic supplementary material:**

The online version of this article (10.1186/s12881-017-0517-1) contains supplementary material, which is available to authorized users.

## Background

Non-ketotic hyperglycinemia (NKH; OMIM 605899), also known as glycine encephalopathy, is an autosomal recessive metabolic disorder caused by a deficiency in the glycine cleavage system (GCS), resulting in a massive accumulation of glycine in body fluids [1]. Most patients present with lethargy and hypotonia in the first week of life, and often progress to apnea requiring ventilation [2]. Some patients die during the neonatal period. The majority of survivors exhibit severe mental retardation and intractable seizures typical of severe NKH [3]. One-sixth of NKH patients have an attenuated form of the disease, half of whom present in early-to-mid infancy with seizures, hypotonia, and developmental delay and/or cognitive impairments, behavioral problems, and impaired work or school performance [4]. Diagnosis is based on the detection of elevated glycine concentrations in cerebrospinal fluid (CSF) together with an increased CSF/plasma glycine ratio.

The GCS consists of the enzymes glycine decarboxylase (P-protein), amino-methyltransferase (T-protein), hydrogen carrier protein (H-protein), and dihydrolipoamide dehydrogenase (L-protein) [5]. The P, T, and H proteins are encoded by *GLDC* (OMIM 238300), *AMT* (OMIM 238310), and *GCSH* (OMIM 238330) genes, respectively. Approximately 70~75% of affected individuals carry disease-causing mutations in *GLDC* gene, whereas 20% and <1% NKH patients have mutations in *AMT* and *GCSH* genes, respectively [6]. In addition, approximately 5% of patients with enzyme-proven glycine encephalopathy do not have a pathogenic variant in *GLDC*, *AMT* or *GCSH* genes; these cases therefore represent a variant form of NKH [7]. Although forming only a relatively small percentage of NKH cases, in a populous country, such as China, it is possible that NKH is much more prevalent in China than it has been realized. Only a few studies have, however, examined the genetic profile of NKH patients in China, with the result that only two Chinese NKH patients have been diagnosed using molecular genetic analyses [8, 9]. We here report the clinical and genetic features of a Chinese family including three siblings with NKH.

## Methods

### Subjects

**Fig. 1:**
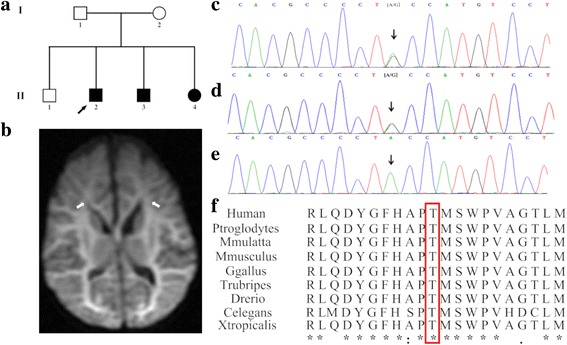
**a** Pedigree of the family. The filled black symbols represent the affected members and the arrow denotes the proband. **b** Diffusion Weight Imaging (DWI) of MRI showed extensive white matter diffusion restriction extending to the subcortical white matter in the proband’s younger sister. White matter shows a hyper-intense signal by DWI. **c-e** Sequence analysis of *GLDC* gene separately identified the heterozygous c.2680A > G variant in the proband (**c**) and his mother (**d**), but not in his father (**e**). **f** Amino acid alignment of the P-protein from several organisms. The position of Thr894 residue (highlighted by a red box) was highly conserved among different species

This study investigated a two-generation Chinese family containing six members of Han ethnicity (Fig. 1a). Moreover, a total of 100 healthy newborns with normal results of neonatal disease screening by tandem mass spectrometry from our center were recruited as controls, these control groups with normal phenotype and have no family history of inherited metabolic diseases including non-ketotic hyperglycinemia. The study was approved by the ethics committee of The Women’s and Children’s Hospital of Quanzhou. All the parents of the patients and 100 control subjects signed written informed consent to participate in the study, and using their own and children’s genetic data, clinical details and/or any accompanying images for scientific research and publication.

### DNA extraction and Sanger sequencing

Genomic DNA was extracted from peripheral whole blood or dried blood spots obtained from the proband and his family members, as well as control subjects. The coding region and flanking intron sequences of *GLDC* (NM_000170.2), *AMT* (NM_000481.3) and *GCSH* (NM_004483.4) genes were amplified using standard PCR (polymerase chain reaction) conditions and bi-directional DNA sequencing. All the primers used are listed in Additional file 1: Table S1. The PCR cycle consisted of an initial denaturation step of 2 min at 95 °C followed by 36 cycles of 30 s at 95 °C, 1 min at 60 °C, and 1 min at 72 °C, and a final step at 72 °C for 2 min. All PCR products were separated and then directly sequenced using BigDye Terminator v.3.1 Mix (Applied Biosystems, Foster City, CA) and analyzed by capillary electrophoresis using an ABI Prism 3500 Genetic Analyzer (Applied Biosystems).

### Multiplex ligation-dependent probe amplification (MLPA)

Deletions or duplications in *GLDC*,*AMT* and *GCSH* genes were analyzed using MLPA [SALSA MLPA P209 Glycine Encephalopathy probe mix (MRC-Holland)]. The analysis was employed according to the manufacturer’s protocol. The MLPA data were analyzed using GeneMarker (version 1.6) software in order to determine potential CN (copy number) variations of exons. The fluorescent signals were compared to normal controls, resulting in a ratio of 0.5 for deletions and 1.5 for duplications. Each test was repeated twice in order to confirm the results.

### Quantitative PCR (Q-PCR)

To verify the variants identified by MLPA, Q-PCR was performed by amplifying *GLDC* exon 3 with two primers sets (Additional file 1: Table S1). The primer sets, referred to as Target 1 and Target 2, were designed to amplify the 3′ and 5′ ends of exon 3 respectively, with the products size being 80-150 bp. The *TERT* gene was chosen as the endogenous control in this study. All reactions using SYBR Green Dye were run using the following cycle: 2 min at 50 °C, 5 min at 95 °C and 40 cycles of 15 s at 95 °C and 30 s at 60 °C. All reactions were performed using the ABI StepOne real-time PCR system. After Q-PCR was performed, the data was collected and analyzed by the 2^-ΔΔCT^ method. Data using Target 1 and Target 2 primers giving a CN = 1 indicated deletion of exon3 of *GLDC* gene.

### In silico analytical tools

The identified variant was checked for its presence in disease databases such as the Human Gene Mutation Database (HGMD) [10], ClinVar [11] and the Leiden Open Variation Database (LOVD) [12], following which several bioinformatic programs (PolyPhen 2, SIFT, PROVEAN and MutationTaster) were employed to predict the impact of a missense change on the protein structure and function [13–16] (Additional file 2: Table S2). Additionally, multiple amino acid sequences were extracted from National Center for Biotechnology Information (NCBI) and aligned to verify the evolutionary conservation using ClustalX (http://www.clustal.org/clustal2) [17, 18].

## Results

### Clinical data and auxiliary examination

Three patients (two males, one female), from one kindred in the Quanzhou area, Fujian Province, southeastern China, were diagnosed with NKH based on their clinical manifestations and an abnormal metabolic profile (Fig. 1a). The non-consanguineous parents are healthy individuals, and their first child was not found to have any clinical symptoms during follow-up. All three affected siblings were born at term with normal birth weight after an uneventful delivery, however, they presented with lethargy, seizures and hypotonia. Both the proband and the younger brother presented with severe clinical symptoms and died during the neonatal period, while the proband’s younger sister, who exhibited relatively milder symptoms, is currently aged 7 months, and has intractable seizures and a profound developmental delay, as shown by magnetic resonance imaging (MRI) (Fig. 1b). Sodium benzoate and dextromethorphan were prescribed for the patient to improve her alertness and decrease seizure frequency. Detailed clinical and biochemical information, as well as the genotypes of the siblings are shown in Table 1.

**Table 1:** Clinical, biochemical and genetic characteristics of the siblings

symbol	Age of onset	Gender	Clinical features	DBS glycine^a^ (μmol/l)	Plasma glycine^b^ (μmol/l)	CSF glycine^c^ (μmol/l)	CSF/plasma ratio^d^	Genotype	Evolution
II-1	Normal	M	Normal	ND	ND	ND	ND	c.2680A > G	Alive at 6y
II-2^e^	Neonatal (2nd day)	M	Lethargy hypotonia seizures apnea	1711.17	ND	ND	ND	c.2680A > G/ Exon 3 Deletion	Died at 11d
II-3	Neonatal (2nd day)	M	Lethargy hypotonia seizures apnea hiccup	1226.96	1587.87	260.2	0.164	c.2680A > G/ Exon 3 Deletion	Died at 13d
II-4	Neonatal (3nd day)	F	Lethargy hypotonia seizures hiccup	971.01	1038.25	157.2	0.151	c.2680A > G/ Exon 3 Deletion	Alive at 7 m; Severe mental retardation, frequent seizures

*ND* Not detected, *d* Day, *m* Month, *y* Year, *F* Female, *M* Male

^a^normal range 178-900 μmol/l

^b^normal range 232-740 μmol/l

^c^normal range 2.2-14.2 μmol/l

^d^normal range < 0.08

^e^the proband

### Mutation identification and bioinformatic analysis

**Fig. 2:**
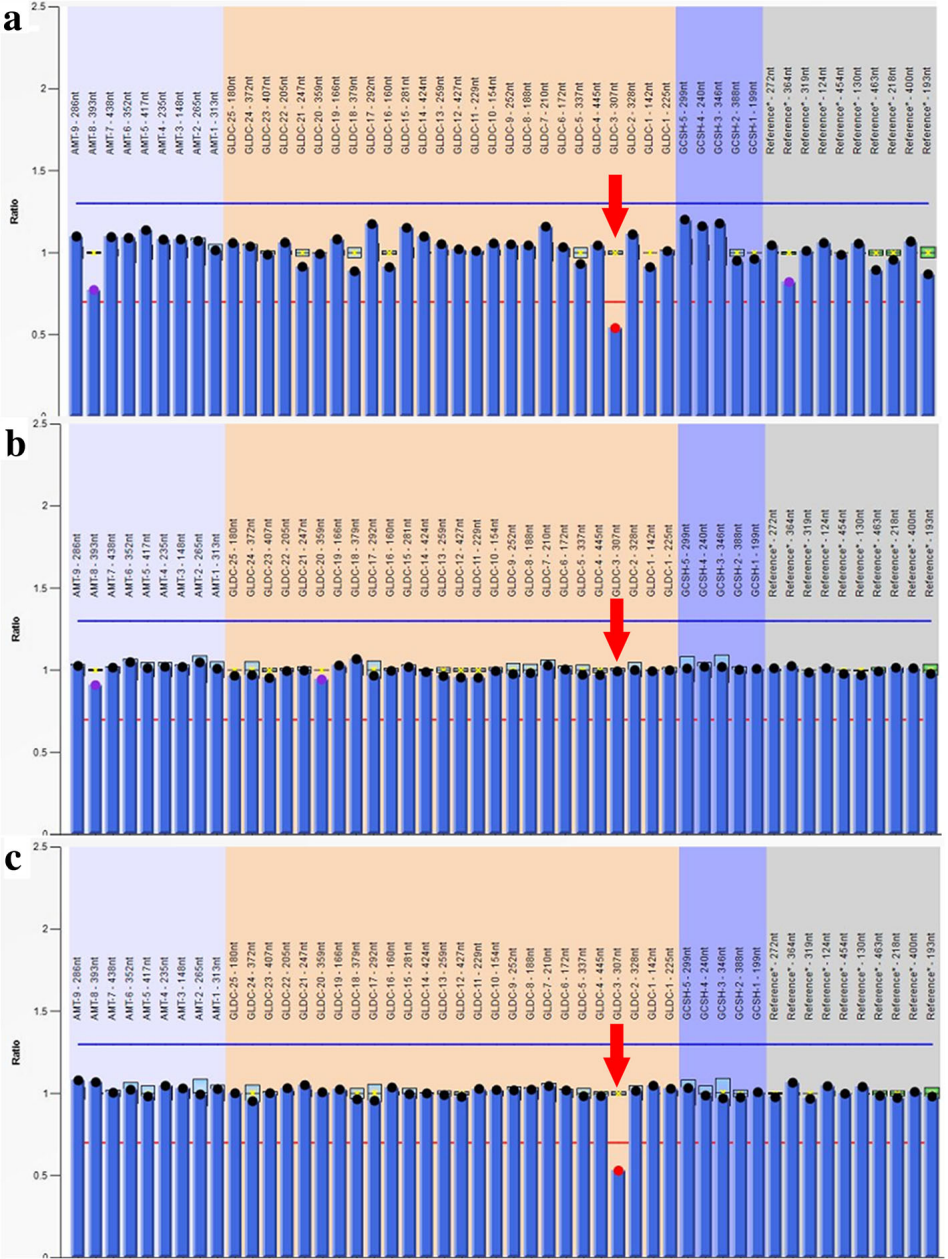
Multiplex ligation-dependent probe amplification analysis of *GLDC*, *AMT* and *GCSH* genes. A heterozygous deletion of exon 3 in *GLDC* gene was detected in the proband (**a**) and his father (**c**), but not in his mother (**b**)

**Fig. 3:**
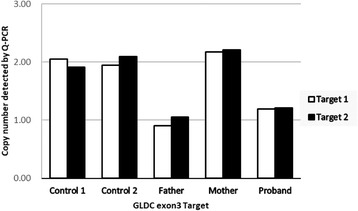
Copy number variations analysis associates exon 3 of *GLDC* gene detected by Q-PCR. Two independent primers were used, the results of which are labeled as Target 1 and Target 2 respectively. The proband and his father both having a CN = 1 indicated a heterozygous deletion of exon 3, and his mother had a CN = 2, identical to the control samples, indicating a normal copy numbers of exon 3

Sanger sequencing identified a novel c.2680A > G variant in exon 23 of *GLDC* gene in the proband (Fig. 1c). This novel variant has not yet been reported in the literature, and was not found in the 1000 Genome, ESP6500, ExAc or dbSNP databases, and, in addition, could not be detected in 100 healthy individuals. In silico analysis predicted that the novel variant was a deleterious mutation ((Additional file 2: Table S2). Additionally, the Thr residue at position 894 was found to be a highly conserved amino acid residue among different species, as assessed using ClustalX software (Fig. 1f), and so a variant of this residue is likely to be deleterious. MLPA analysis combined with Q-PCR experiments also revealed an exonic deletion event in the proband, namely a heterozygotic deletion of exon 3 (Figs. 2 and 3).

These findings indicated that the proband had compound heterozygous variants in *GLDC* gene, i.e. a c.2680A > G (p.Thr894Ala) variant in exon 23 and a heterozygous deletion of exon 3, which were inherited respectively from the mother and the father (Figs. 1d, e and 2). The same variants were observed in the proband’s younger brother and sister, whereas his unaffected elder brother carried only the maternal variant.

## Discussion

Clinically, the patients in this Chinese family are representative of neonatal NKH patients. The proband, along with his younger siblings, exhibited severe and progressive manifestation such as lethargy, hypotonia and seizures, with greatly elevated glycine levels in their plasma and CSF. Molecular genetic analysis by Sanger sequencing and MLPA, identified compound heterozygous variants in *GLDC* in these three patients, namely a c.2680A > G (p.Thr894Ala) variant in exon 23 and a heterozygous deletion of exon 3, which were inherited respectively from their parents. Although these three siblings have the same compound heterozygous variants in *GLDC* gene, and similar clinical phenotypes, the severity of the disease, as well as the outcome, were variable. Because of this, we speculate that other genetic and environmental factors may also be responsible for the clinical phenotype including the severity and the outcome, which requires further research.

NKH is primarily caused by mutations in *GLDC* gene. The 113.15 kb *GLDC* gene located on chromosome 9p24.1, encodes a 1021 amino acid protein called glycine decarboxylase. To date, more than 122 mutations in *GLDC* have been reported to cause NKH, including a multitude of missense mutations and different deletions involving multiple *GLDC* exons [19]. The mutations found in NKH are highly heterogeneous, although recurrent missense mutations reported include the p.R515S mutation found in Caucasians and the p.S564I mutation observed in Finnish populations [6, 20]. Intragenic copy number variations (CNVs) have been noted in approximately 20% of *GLDC* alleles, the majority of which are multi-exon deletions [21]. These mutated alleles caused by multi-exon deletions or duplications occur in various haplotypes and in different ethnic groups. In China, to the best of our knowledge, only two NKH patients with compound heterozygous mutations in *GLDC* gene have been reported to date [8, 9].

In this work, we describe the novel variant c.2680A > G, which leads to the substitution of the polar amino acid threonine with the nonpolar animo acid alanine at position 894 of the P-protein. Computational analysis predicted that the variant is likely to have pathogenic significance and a conservation analysis in different species showed that this amino acid was highly conserved across a broad range of species, which again strongly suggests that the variant at this site might be deleterious. The deletion of exon 3 of the *GLDC* gene is a frameshift variant resulting in the premature termination of the P-protein, and is also likely to be clinically significant. This CNV has previously been reported by Coughlin et al. in a systematic study of 578 families, among which one child had a homozygous deletion of exon 3 [22], and this is the first time this variant has been found in the Chinese population. Although the MLPA technique fails to pinpoint the exact location of this deletion, based on MLPA probes and subsequent qPCR experiments, we know that the upstream breakpoint was from the MLPA probe binding site of exon 2 to qPCR upstream primer 5′ site of exon 3, and the downstream breakpoint was from qPCR downstream primer 3′ site of exon 3 to the MLPA probe binding site of exon 4 (Additional file 3: Figure S1). According to the HGVS nomenclature, the break point can be expressed as NG_016397.1 (NM_000170.2): c. (261_335-95) _ (470 + 111_476) del. In short, in view of the fact that this CNV might be overlooked by conventional sequencing strategies, MLPA or another technique that detects deletions should therefore also be utilized to analyze the presence of potential CNVs in *GLDC* gene.

## Conclusions

In this study, we describe the clinical and genetic features of a Chinese family with three siblings affected with NKH. A novel variant, as well as a previously reported deletion in *GLDC* gene, were identified in the three affected siblings. Thus, our findings suggest that these two variants in *GLDC* gene probably underlie the pathogenesis of NKH in this family, and also enrich the mutational spectrum of *GLDC* gene.

## Additional files

Additional file 1: Table S1. List of the primers used for Sanger sequencing and Q-PCR (DOCX 13 kb)

Additional file 2: Table S2. Pathogenicity prediction analysis of *GLDC* c.2680A > G alteration (DOC 29 kb)

Additional file 3: Figure S1. The illustration of breakpoints in exon 3 deletion (TIFF 891 kb)

## Abbreviations

CN: Copy number
CNV: Copy number variation
CSF: Cerebrospinal fluid
GCS: Glycine cleavage system
MLPA: Multiplex ligation-dependent probe amplification
MRI: Magnetic resonance imaging
NKH: Non-ketotic hyperglycinemia
PCR: Polymerase chain reaction
