# Supplementary material for: A novel compound heterozygous variant identified in GLDC gene in a Chinese family with non-ketotic hyperglycinemia
Source: BMC Med Genet. 2018 Jan 5;19:5. doi: 10.1186/s12881-017-0517-1 (PMC5755286; doi:10.1186/s12881-017-0517-1)
Supplement: Supplementary file 1 — List of the primers used for Sanger sequencing and Q-PCR (DOCX 13 kb) [file 12881_2017_517_MOESM1_ESM.docx]

**Additional file 1: Table 1. List of the primers used for Sanger sequencing and Q-PCR**

| Gene | Exon | Forward Sequence (F) |
| --- | --- | --- |
|  |  | Reverse Sequence (R) |
| *GLDC*(Sanger) | exon 1 | F: AAACGACGGCCAGTGGGACTCCACGG |
|  |  | R: AACAGCTATGACCGCACGAGGACCA |
|  | exon 2 | F: AACGACGGCCAGTGTAGTCGCAAAACA |
|  |  | R: AACAGCTATGACCTAATCCACACATT |
|  | exon 3 | F: AACGACGGCCAGTTTAGTGTTGGTTGG |
|  |  | R: AACAGCTATGACCGTGGGTGTCAGTGT |
|  | exon 4 | F: AACGACGGCCAGTCCCAAACAAAGG |
|  |  | R: GCTATGACCTGTTGAGTTTTTCAGTTT |
|  | exon 5 | F: AAACGACGGCCAGTGTGCTTAGACTTA |
|  |  | R: ACAGCTATGACCCTGTTTCAGATT |
|  | exon 6-7 | F: AACGACGGCCAGTTGACAGGAAA |
|  |  | R: CAGCTATGACCGCGTCCATCAATCT |
|  | exon 8 | F: AACGACGGCCAGTTGCTGTGATTATC |
|  |  | R: AAACAGCTATGACCAGGTGGTGAATAAAT |
|  | exon 9 | F: ACGACGGCCAGTTGTTTTGTCGCTG |
|  |  | R: AAACAGCTATGACCTTCCTCGTTTCTCA |
|  | exon 10 | F: ACGACGGCCAGTTACACCAAAATACACGC |
|  |  | R: ACAGCTATGACCGTCTAAAAGAAAG |
|  | exon 11 | F: ACGACGGCCAGTTCTCCCCTTTGA |
|  |  | R: AACAGCTATGACCCCCTTCTCCCTCA |
|  | exon 12 | F: AACGACGGCCAGTAGGATTGAGCCCA |
|  |  | R: ACAGCTATGACCGCTGAGCCAGAATA |
|  | exon 13-14 | F: ACGACGGCCAGTCTCATACCCACA |
|  |  | R: AACAGCTATGACCGATAGAAAGGCATAA |
|  | exon 15 | F: AACGACGGCCAGTACAACTATGTCCC |
|  |  | R: AAACAGCTATGACCCAAGTCACAGAAT |
|  | exon 16 | F: ACGACGGCCAGTCTGGATGCTGTTGC |
|  |  | R: AACAGCTATGACCAGGCTTGGAGGGA |
|  | exon 17 | F: ACGACGGCCAGTTCAGCCTAAATAA |
|  |  | R: AACAGCTATGACCATAATCCATCA |
|  | exon 18 | F: AACGACGGCCAGTTTTGTGAAGGGAGT |
|  |  | R: AAACAGCTATGACCGTCCCCCAAGTAA |
|  | exon 19 | F: AAACGACGGCCAGTTGCCTGCTTTTC |
|  |  | R: AAACAGCTATGACCACCCATTTTGCTGC |
|  | exon 20 | F: AACGACGGCCAGTCTCTGCTAAGAAC |
|  |  | R: AAACAGCTATGACCCACTCTGAGAACCC |
|  | exon 21 | F: ACGACGGCCAGTGTGGAGTTTATCTG |
|  |  | R: AAACAGCTATGACCTATGTGCTAAGAAATAGA |
|  | exon 22 | F: AACGACGGCCAGTTTGGTCCTCGCTG |
|  |  | R: AACAGCTATGACCAGTCATAAGCAAACTTT |
|  | exon 23 | F: AAACGACGGCCAGTTCTGATGATTTTCTTA |
|  |  | R: CAGCTATGACCAGTTGAGAGTTCGGG |
|  | exon 24 | F: GCCAGTGAAGATTGACGGTTGA |
|  |  | R: AAACAGCTATGACCCTGTGGTTGATACTG |
|  | exon 25 | F: TGGTAATGTAGGG |
|  |  | R: GATGTAACAATGGC |
| *AMT*(Sanger) | exon 1-2 | F: AACGACGGCCAGTTCTGGAGACAGGG |
|  |  | R: AAACAGCTATGACCGTGTCAGCCCTGGAA |
|  | exon3 | F: AAACGACGGCCAGTGTCAGGAAATCAGA |
|  |  | R: AACAGCTATGACCGAGCAGAAATAAA |
|  | exon 4 | F: AACGACGGCCAGTAAGCCAAAACGC |
|  |  | R: AAACAGCTATGACCGTAATCCCCCAC |
|  | exon 5-7 | F: AACGACGGCCAGTCAGAACTGGGCTTG |
|  |  | R: AACAGCTATGACCGAAGGCTTCAGGCTA |
|  | exon 8-9 | F: ACGACGGCCAGTCAGGGGATAGGAGGTG |
|  |  | R: AACAGCTATGACCCATTGGGTTGGCAT |
| *GCSH*(Sanger) | exon 1 | F: CGACGGCCAGTCCAGTCAGGCTCTG |
|  |  | R: AACAGCTATGACCGGGTCCTCCCGCTA |
|  | exon 2 | F: AACGACGGCCAGTAGTGTCAACAGGTC |
|  |  | R: AACAGCTATGACCGTAGAGATAAAGCAA |
|  | exon 3 | F: ACGACGGCCAGTAAACTCAAGAATGTAG |
|  |  | R: AACAGCTATGACCGGTGACAAAGCAA |
|  | exon 4 | F: AACGACGGCCAGTCAGAGCGAGACTC |
|  |  | R: ACAGCTATGACCAAGAAGTAGAAAAAGAT |
|  | exon 5 | F: ACGACGGCCAGTTCCCCTTCTCGGGTT |
|  |  | R: ACAGCTATGACCCTACTTGGAAATAACTTTTGG |
| *GLDC*(Q-PCR) | exon 3  -Target 1 | F: ATGTATTTCTCAGTT |
|  |  | R: GGAGGTGGGTGTCA |
|  | exon 3  -Target 2 | F: TAAATACTCAAACAAA |
|  |  | R: AAAAAGAGAAATGTT |
| *TERT*(Q-PCR reference gene) |  | F: GTTCAGGGCACACGATTGGT |
|  |  | R: CTGGCCTCCTTTGCGATC |
