# Supplementary material for: A novel compound heterozygous variant identified in GLDC gene in a Chinese family with non-ketotic hyperglycinemia
Source: BMC Med Genet. 2018 Jan 5;19:5. doi: 10.1186/s12881-017-0517-1 (PMC5755286; doi:10.1186/s12881-017-0517-1)
Supplement: Supplementary file 2 — Pathogenicity prediction analysis of GLDC c.2680A > G alteration (DOC 29 kb) [file 12881_2017_517_MOESM2_ESM.doc]

**Additional file 2: Table S2.** Pathogenicity prediction analysis of *GLDC* c.2680A>G alteration

| Bioinformatic program | URL | Score | Prediction effect |
| --- | --- | --- | --- |
| PolyPhen-2 | <http://genetics.bwh.harvard.edu/pph2/> | 0.999 | Probably damaging |
| SIFT | <http://sift.jcvi.org/> | 0.000 | Not tolerated |
| PROVEAN | <http://provean.jcvi.org/index.php> | -3.65 | Deleterious effect |
| MutationTaster | <http://www.mutationtaster.org/> | 1.000 | Disease causing |
