# Supplementary figures and images for: A novel compound heterozygous variant identified in GLDC gene in a Chinese family with non-ketotic hyperglycinemia
Source: BMC Med Genet. 2018 Jan 5;19:5. doi: 10.1186/s12881-017-0517-1 (PMC5755286; doi:10.1186/s12881-017-0517-1)

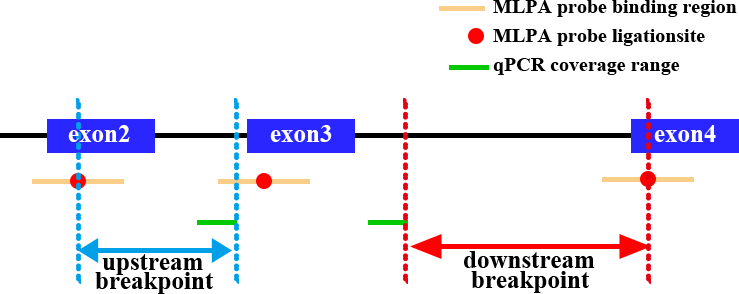

Supplement: Supplementary file 3 — The illustration of breakpoints in exon 3 deletion (TIFF 891 kb) [file 12881_2017_517_MOESM3_ESM.tif]
